# Account of Diseases in an Eastern District of London

**Published:** 1802-05

**Authors:** 


### ACCOUNT OF DISEASES IN AN EASTERN DISTRICT OF LONDON,

*From March* 20, *to April* 20, 1802.

| | |
|---|---|
| **ACUTE DISEASES.** | |
| Typhus | 7 |
| Pneumonia | 3 |
| **CHRONIC DISEASES.** | |
| Catarrh | 8 |
| Phthiſis Pulmonalis | 3 |
| Tuſſis | 19 |
| Dyſpnœa | 13 |
| Tuſſis cum Dyſpnœa | 11 |
| Hydrothorax | 1 |
| Pleurodynia | 4 |
| Anaſarca | 3 |
| Palpitatio | 2 |
| Apoplexia | 1 |
| Paralyſis | 2 |
| Cephalalgia | 8 |
| Ophthalmia | 4 |
| Gaſtrodynia | 3 |
| Dyſpepſia | 12 |
| Vomitus | 3 |
| Conſtipatio | 4 |
| Enterodynia | 3 |
| Hæmorrhois | 3 |
| Fluor Albus | 5 |
| Amenorrhœa | 7 |
| Rheumaticus Chronicus | 15 |
| **PUERPERAL DISEASES.** | |
| Ephemera | 4 |
| Dolores Poſt Partum | 2 |
| Abſceſſus Mammæ | 1 |
| **INFANTILE DISEASES.** | |
| Rubeola | 8 |
| Pertuſſis | 7 |
| Ophthalmia Purulenta | 1 |

The diſeaſes of children continue to engage conſiderable attention. The meaſles and hooping cough are ſtill prevalent amongſt patients of this claſs. The meaſles, in general, appear in a milder form at preſent, though, in ſome inſtances, the diſeaſe is ſtill attended with a conſiderable degree of thoracic affection; the cough continues for ſome time after every ſign of the eruption has ceaſed, and in ſome patients ſeems to threaten a more ſerious affection of the lungs.

The hooping cough is ſtill very prevalent; a conſiderable degree of fever frequently attends it, and even where the ſymptoms have been mild, the long continuance of them leaves the patient in a very feeble ſtate. As the ſpring is advancing, a removal into the country, moderate exerciſe in the air, with the uſe of aſſes milk, and other articles of diet of a mild and nutritious nature, may afford conſiderable relief.

CRITICAL

